# Daptomycin treatment in patients with resistant staphylococcal periprosthetic joint infection 

**DOI:** 10.1186/s12879-017-2842-6

**Published:** 2017-11-29

**Authors:** Yu-Jui Chang, Mel S. Lee, Chen-Hsiang Lee, Po-Chun Lin, Feng-Chih Kuo

**Affiliations:** 1 Department of Orthopedic Surgery, Kaohsiung Chang Gung Memorial Hospital, No 123, Ta Pei Road, Niao Sung Dist, Kaohsiung, Taiwan; 2 College of Medicine, Chang Gung University, Kaohsiung, Taiwan; 3 Division of Infectious Diseases, Department of Internal Medicine, Kaohsiung Chang Gung Memorial Hospital, Kaohsiung, Taiwan

**Keywords:** Daptomycin, Prosthetic joint infection, *Staphylococcus aureus*, Resistant

## Abstract

**Background:**

Resistant staphylococcal organisms remain a serious problem in the treatment of periprosthetic joint infection (PJI). Higher failure rates have been reported when vancomycin was used. The purpose of this study was to assess the clinical dosage, effect, and safety of daptomycin in patients with resistant staphylococcal PJI.

**Methods:**

We retrospectively enrolled patients with hip or knee PJI who were treated with daptomycin in our institution (*n* = 16) from January 2013 to December 2014 with a minimum follow-up of 2 years. The patients received daptomycin when glycopeptide could not be used due to multiple resistance, any adverse reaction, chronic kidney disease stage 3 or worse, and previous treatment failure with glycopeptide or empirical therapy.

**Results:**

These patients received daptomycin at a median dose of 8.3 mg∕kg per day for a median duration of 14 days. The overall treatment success rate was 87.5% (14 of 16 cases) after a median follow-up period of 27 months. In the subgroups of acute and chronic PJI, the success rate was 80% and 91%, respectively. One patient developed asymptomatic transient serum aspartate transaminase (AST) elevation. No severe side effects such as myositis, acute renal failure due to rhabdomyolysis or eosinophilic pneumonia were found in our series.

**Conclusion:**

Relatively high daptomycin doses combined with adequate surgical intervention were effective in treating resistant staphylococcal PJI. Daptomycin is an option worthy of consideration in PJI patients for whom glycopeptide treatment is unsuitable. Further prospective randomized comparative study is needed in the future.

## Background

Periprosthetic joint infection (PJI) is a serious complication related to significant morbidity, mortality, and medical costs [1]. The incidence of PJI has been reported to be around 0.5% to 1.0% for hip replacement cases and 0.5% to 2% for knee replacements [2]. The pathogenesis of PJI is mainly attributable to the formation of a biofilm caused by microorganisms attaching to the surface of the involved prosthesis, the biofilm being resistant to host defences and antimicrobial agents [3]. Once a biofilm has become established, the difficulty of successful treatment is increased, and removal of the involved prosthesis is necessary in chronic cases [4]. Treatment of PJI is based upon the period during which microorganisms are attached to the prosthetic joint and the formation of an organized biofilm that is attached to the implant [5]. In chronic cases lasting more than 4 weeks, a two-stage reimplantation is the gold standard procedure worldwide [6]; with regards to acute cases lasting fewer than 4 weeks, emergency debridement with exchange of polyethylene and prosthesis retention is an acceptable alternative treatment [7]. The most important strategy for the treatment of either type of PJI is to combine adequate surgical intervention and appropriate antibiotic therapy.

Gram-positive cocci (*Staphylococcus aureus* and coagulase-negative Staphylococci) is the leading microorganism in PJI [8]. Furthermore, the incidence rate of PJI caused by methicillin-resistant Staphylococci is a rising concern [9]. Patients with resistant staphylococcal PJI are often treated with a glycopeptide such as vancomycin or teicoplanin. Higher treatment failure was noted when intravenous vancomycin had been administered in cases of resistant staphylococcal PJI with minimum inhibitory concentrations (MICs) > 1.5 mg/L [10, 11].

Daptomycin is a newer option for the treatment of PJI owing to its excellent bactericidal activity against gram-positive bacteria, especially MDR strains [12]. The modest advantage of daptomycin over other drugs reflects the presence of a higher fraction of surface or near-surface organisms in an in vitro model; these organisms would be expected to be remain susceptible to the rapid cidal activity of daptomycin [13]. Furthermore, daptomycin penetrates bone effectively and disrupts multiple bacterial plasma membrane functions without penetrating the cytoplasm [14]. The clinical efficacy and safety of daptomycin have been proven in patients with renal impairment, especially patients with vancomycin-associated nephrotoxicity [15]. However, few studies have investigated daptomycin as a possible option for the treatment of resistant staphylococcal PJI [16, 17]. We believe daptomycin to be effective and well-tolerated in patients with PJI caused by resistant staphylococcal organisms. The study aimed to review clinical practice in terms of daptomycin treatment, with specific emphasis on its clinical outcome, safety, and tolerability in patients with resistant staphylococcal PJI.

## Methods

We retrospectively enrolled patients with hip or knee PJI who were treated with daptomycin in our institute from January 2013 to December 2014 with a minimum follow-up of 2 years. We recorded patient demographics, comorbidities, the estimated glomerular filtration rate (eGFR) [18], the location of the prosthesis, type of PJI, surgical methods, microbiological results, dosages and treatment duration, in addition to the reason for daptomycin treatment, its side effects and clinical efficacy. All patients were classified based on the Tsukayama classification [5], which categorizes PJI according to the duration from prosthesis implantation.

Standard protocols for PJI treatment were adopted. For type II and type III acute infection, urgent surgical debridement with exchange of mobile parts and prosthesis retention were performed, followed by systemic antibiotic therapy for 4−6 weeks. In type IV chronic infection, a two-stage reimplantation protocol was adopted as previously described [19]. In the first stage, the operative procedure included removal of the implant, aggressive debridement of the joint and insertion of a high-dose, antibiotic-loaded cement spacer or beads for topical antibiotic delivery. To effectively target the causative pathogen and deliver antibiotic treatment, joint synovial fluid was collected in a blood culture bottle, in addition to 3 to 4 sets of tissue cultures. The causative microorganism was confirmed if at least two positive samples of the same microorganism were identified or matched to blood, joint synovial fluid, or tissue culture. After the culture results were known, an infectious disease specialist was consulted to recommend appropriate antibiotics.

Daptomycin was indicated if the patient’s condition met any one of the following criteria: glycopeptide antibiotics could not be used due to resistance or any adverse reaction such as allergy or phlebitis; vancomycin MIC > 1.5 mg/L; previous treatment failure with intravenous glycopeptide; empirical therapy in cases of suspected MDR Gram-positive cocci PJI; and chronic kidney disease (CKD) stage 3 or worse (eGFR < 60 mL∕min∕1.73m^2^) [16]. Daptomycin treatment in every case was initiated after consultation with infectious disease specialists, and the dosage and duration were also based on specialist guidance. Based on the recent Infectious Diseases Society of America guidelines and clinical reports [16, 17, 20], daptomycin may be administered as an alternative option to manage resistant staphylococcal PJI at a dose ≥ 6 mg/kg per day. In patients with advanced renal insufficiency (stage 4 or worse), daptomycin should be administered every 48 h [21].

All patients had received oral antibiotics following parenteral antibiotics after discharge. The median period of oral antibiotic treatment after discharge was 35 days (range: 6−65 days). The oral antibiotic combinations included sulfamethoxazole/trimethoprim and rifampin or fusidic acid and rifampicin. The criteria for reimplantation surgery included a reduced erythrocyte sedimentation rate (ESR), return to near-normal C-reactive protein (CRP) level, and a satisfactory wound status. All reimplantations were performed after a 2-week antibiotic holiday without elevation of ESR and CRP. After prophylaxis with intravenous 1 g vancomycin, new prostheses were reimplanted with 1 g daptomycin in a pack of 40 g of bone cement [Stryker Orthopaedics, Mahwah, New Jersey] for knee or hip prosthesis fixation if cement fixation was needed in the second stage. After reimplantation, the patients received systemic antibiotics until the intraoperative culture results showed negative finding. No further oral antibiotics were administered after discharge.

A successful clinical outcome after daptomycin therapy was defined as resolution of clinical signs and symptoms and/or no prolonged suppressive oral antibiotic treatment, and CRP and ESR levels that had returned to the normal range at the last follow-up. Failure was defined as an inadequate response to therapy, worsening or new/recurrent signs and symptoms, the need for a change of parenteral antibiotic therapy or prolonged suppressive oral antibiotic treatment, a positive culture at the end of therapy, or the requirement for re-operation [22]. Prolonged suppressive oral antibiotic treatment was defined as oral antibiotic therapy prescribed for a duration longer than 6 months [23]. Patients were assessed weekly for daptomycin adverse effects following initiation of daptomycin treatment, including serum creatine phosphokinase (CPK), liver enzymes, and other associated blood parameters. Other adverse effects, including low blood pressure, high blood pressure, swelling, insomnia, rash, diarrhea, abdominal pain, eosinophilia and eosinophilic pneumonia, dyspnea, fever, hypersensitivity, myopathy and rhabdomyolysis, were also monitored.

## Results

Sixteen patients were treated with daptomycin for resistant staphylococcal PJI during the study period and completed follow-up for at least 2 years; one patient was excluded due to loss to follow-up within 6 months. The median age of the 16 patients was 66.5 years (range: 52−86 years). The types of infection were as follows: 5 patients with acute infection (type II and III) who had received surgical debridement and implant retention; and 11 patients with chronic infection (type IV) who had received two-stage reimplantation (Table 1). Methicillin-resistant *Staphylococcus aureus* (MRSA) accounted for 62.5%, and methicillin-resistant coagulase-negative Staphylococci (MRCoNS) for 37.5%. The reasons for using daptomycin included vancomycin MIC > 1.5 mg∕L in 1 case, previous glycopeptide failure in 2 cases, impaired renal function in 2 cases, empirical treatment in 4 cases, and adverse effects such as phlebitis or allergy in 7 cases (Table 2). Daptomycin was administered at a dose range of 3.3−10.6 mg/kg per day according to the infectious disease specialists’ suggestions. The median dosage of daptomycin was 8.3 mg/kg per day, and the median treatment duration was 14 days (Table 2). Debridement and prosthesis retention in the acute infection group was successful in 4 cases (80%) but failed in 1 case (20%), while a two-stage surgical protocol for chronic PJI was successful in 11 cases (91%) but failed in 1 case (9%) (Table 2).

**Table 1:** Characteristics of patients with resistant staphylococcal periprosthetic joint infection

Patient	Weight (Kg)	Prosthesis type	Tsukayama classification^a^	Surgical procedure
1	50	TKA	IV	two-stage reimplantation
2	60	TKA	IV	two-stage reimplantation
3	77	TKA	IV	two-stage reimplantation
4	60	THA	II	DAIR^b^
5	65	TKA	IV	two-stage reimplantation
6	47	THA	II	DAIR^b^
7	60	TKA	IV	two-stage reimplantation
8	70	TKA	IV	two-stage reimplantation
9	50	TKA	III	DAIR^b^
10	79	THA	IV	two-stage reimplantation
11	50	THA	IV	two-stage reimplantation
12	75	THA	IV	two-stage reimplantation
13	68	TKA	III	DAIR^b^
14	60	THA	IV	two-stage reimplantation
15	61	TKA	II	DAIR^b^
16	47	THA	IV	two-stage reimplantation

^a^DAIR: debridement, antibiotics, irrigation, prosthesis retention

^b^Tsukayama classification system: type I, intraoperative positive culture; type II, acute infections; type III, hematogenous infections; type IV, chronic infections

**Table 2:** Information regarding patients’ infections and daptomycin usage

Patients	Surgical procedure	Pathogen	DAP Reason	DAP dosage (mg/kg/day)	DAP duration (days)	Total parental antibiotics (days)	Total time under DAP (%)	Adverse effects	Outcome	Follow-up (months)
1	two-stage reimplantation	MRSA	intolerance	10	10	13	77	none	success	26
2	two-stage reimplantation	MRSA	intolerance	8.3	18	35	51	none	success	27
3	two-stage reimplantation	MRCoNS	CKD stage 5	3.3	12	15	80	none	success	25
4	DAIR	MRCoNS	intolerance	8.3	23	23	100	none	success	41
5	two-stage reimplantation	MRSA	CKD stage 3	7.7	13	13	100	none	success	23
6	DAIR	MRSA	teicoplanin failure	10.6	11	21	52	AST elevation	success	25
7	two-stage reimplantation	MRCoNS	intolerance	8.3	12	26	46	none	success	27
8	two-stage reimplantation	MRSA	intolerance	7.2	18	49	37	none	success	24
9	DAIR	MRSA	vancomycin MIC >1.5 mg/L	10	27	32	84	none	success	26
10	two-stage reimplantation	MRSA	empirical	6.3	21	33	64	none	failure	24
11	two-stage reimplantation	MRCoNS	intolerance	10	13	36	36	none	success	34
12	two-stage reimplantation	MRSA	teicoplanin failure	6.7	14	39	36	none	success	36
13	DAIR	MRSA	intolerance	7.4	14	20	70	none	success	29
14	two-stage reimplantation	MRCoNS	empirical	8.3	16	22	73	none	success	36
15	DAIR	MRSA	empirical	8.3	26	66	39	none	failure	34
16	two-stage reimplantation	MRCoNS	empirical	10.6	11	19	58	none	success	42

*DAP* Daptomycin, *MRSA* Methicillin-resistant *Staphylococcus aureus*, *MRCoNS* Methicillin-resistant coagulase-negative staphylococci CKD, chronic kidney disease, *MIC* Minimum inhibitory concentration, *AST* Aspartate aminotransferase; intolerance, allergy to glycopeptide or phlebitis

Among the 16 patients, one developed asymptomatic transient elevation of serum aspartate transaminase (AST) level during the treatment course (Patient 06). In this patient, the AST level elevated from 55 U∕L at baseline to 108 U∕L on day 3 of daptomycin treatment, but rapidly normalized on day 6, while no specific complaint or discomfort was noted, and the ALT and CPK levels were within the normal ranges. No other adverse effects such as eosinophilic pneumonia, massive rhabdomyolysis or acute renal failure were reported in our series.

In two of 4 patients (50%) who underwent empirical daptomycin treatment for suspected MDR Gram-positive cocci PJI after discussion with an infectious disease specialist, treatment failed eventually. One (Patient 15) was a 65-year-old male who developed acute left knee PJI caused by MRSA during the 2 weeks after primary total knee replacement and underwent an urgent debridement operation. After the operation, we initiated daptomycin therapy (8.3 mg∕kg per day). The patient’s CRP and ESR levels remained high after 3 weeks, and left knee synovial fluid examination revealed a high leukocyte count and positive culture results. Thus, a second debridement was performed, and a teicoplanin regimen was implemented for approximately 6 weeks, which was then shifted to oral antibiotics for 3 weeks until the CRP and ESR levels reached the normal ranges. Finally, the patient recovered well, and no recurrent infection occurred during a 34-month follow-up period. The other case of treatment failure (Patient 10) was a 60-year-old male patient who underwent resection arthroplasty of the left hip for chronic PJI. After the surgery, daptomycin therapy (6.3 mg∕kg per day) was instigated, but persistently high CRP and ESR levels with left thigh erythema and pus discharge were noted after 21 days of daptomycin therapy. The treatment was shifted to teicoplanin, and several debridements were performed. After a six-month follow-up period, the patient’s clinical symptoms had improved, and the CRP and ESR levels had reached the normal ranges. Thus, reimplantation surgery was performed, and no recurrent infection occurred within a 24-month follow-up period.

## Discussion

Vancomycin has been considered the first choice parenteral antibiotic for the treatment of resistant staphylococcal PJI [4]; however, vancomycin has not been demonstrated to result in a favorable outcome in patients with methicillin-resistant staphylococcal PJI [24]. Furthermore, a risk of vancomycin-induced nephrotoxicity in the population with chronic kidney disease has also been reported [25]. In a recent meta-analysis study related to MRSA-infected patients, high vancomycin trough levels were recognized as an independent factor associated with risk of nephrotoxicity [26]. For the above reasons, vancomycin is not optimal for the treatment of resistant staphylococcal PJI in patients with CKD. Daptomycin is indicated when vancomycin or teicoplanin cannot be used due to intolerance, allergy, or previous treatment failure, or in patients with poor renal function. In our study, two patients with CKD were successfully treated with daptomycin without acute kidney injury. Daptomycin, at a median dose of 6.0 mg∕kg administered every 24 h or 48 h, showed efficacy and safety in patients with renal impairment [15]. In this study, daptomycin therapy was administered in more than 50% of patients due to baseline renal impairment from prior vancomycin exposure. The authors concluded that daptomycin is a safe and effective therapeutic agent for use in patients with renal impairment for whom previous treatment had failed or in those who cannot tolerate vancomycin.

There were some limitations of our study. First, the lack of randomized comparative information limited the clinical results. Second, the data pool was too small to obtain statistically-significant results. Third, in chronic infection cases, the component of antibiotic-loaded cement spacer or beads used was not the same in each case. Fourth, the duration of oral antibiotic therapy following parenteral antibiotics varied in this study in the first stage, and postoperative oral antibiotics were not prescribed after reimplantation. Oral antibiotics can effectively suppress manifestations of residual infection, and some studies have suggested that postoperative oral antibiotics can effectively reduce the reinfection rate following two-stage revision arthroplasty [27]. Finally, we did not provide information regarding the drugs or the concentrations of antibiotic-impregnated cement routinely used in the two-stage procedure.

The overall success rate of treatment of resistant PJI in this study was 87.5%. In the subgroup of acute PJI cases, treatment with daptomycin, debridement and prosthesis retention was successful in 80% of patients, and for chronic cases, the success rate increased to 91%. The success rate of daptomycin treatment for PJI has varied greatly among different reports in the literature, from 54.5% to 78.6% (Table 3) [16, 17, 21, 28, 29]. The reason for this variability may be related to an inadequate dosage of daptomycin prescribed in some studies. For example, Rao et al. used daptomycin at a median dosage of 4 mg∕kg per day in 11 cases, and achieved a lower success rate of 54.5% [21]. Antony et al. reported a 38% success rate among patients treated with 4 mg∕kg per 24 or 48 h as compared with a 77% success rate among patients who received daptomycin at 6 mg∕kg per day [26]. Byren et al. [17] used daptomycin at 6 or 8 mg∕kg per day for 6 weeks in a randomized trial during the two-stage reimplantation process, and found that the higher-dose group (8 mg∕kg per day) exhibited a higher treatment success rate than the lower-dose (6 mg∕kg per day) group. Other clinical studies also supported the efficacy and safety of higher daptomycin doses up to 8 mg∕kg per day or more [30, 31]. In our study, patients who received an adequate dosage of daptomycin with suitable surgical intervention for the treatment of PJI (median dosage of 8.3 mg∕kg per day) had successful outcomes. However, in 50% of patients (2 of 4) in whom treatment failed when daptomycin was administered for empirical reasons, following shifting to teicoplanin therapy, success was achieved. This would seem to suggest that daptomycin is problematic as a first-line treatment, and that the treatment outcome may be impacted by the initial antibiotic, multiple surgical procedures, and further oral therapy. We believe that daptomycin cannot replace glycopeptide for the treatment of resistant staphylococcal PJI, but it is an option worthy of consideration.

**Table 3:** Data on the clinical daptomycin use in patients with staphylococcal periprosthetic joint infection

Study	No of patients	Daptomycin dose (median, mg/kg/day)	Daptomycin duration (median, days)	Adverse event (%)	Success rate (%)
Antony et al. [29] (2006)	8	6.0	42	6.5	75.0
Rao et al. [21] (2006)	11	4.0	42	NA	54.5
Antony et al. [28] (2009)	30	6.0	37	3.3	66.7
Corona et al. [16] (2012)	14	6.6	44	21.4	78.6
Byren et al. [17] (2012)	24	6.0	42	8.0	58.3
	13	8.0	42	16.7	60.9
Our study	16	8.3	14	6.3	87.5

Daptomycin has been reported to be well-tolerated in several clinical trials with a wide therapeutic dosage window. However, it can occasionally cause adverse effects, such as elevation of liver enzyme and CPK levels, myalgia, rhabdomyolysis, and acute renal failure [32]. In addition, concomitant use of daptomycin and statins carries concern regarding potential synergistic musculoskeletal toxicity [33]. The adverse effects are fewer if a shorter course of systemic daptomycin is prescribed [19]. Regular monitoring of serum creatine and CPK levels along with symptoms of myopathy would be a useful strategy in patients receiving daptomycin treatment. In our short patient series, we observed one case of asymptomatic AST elevation judged as directly associated with daptomycin administration at a dosage of 10.6 mg∕kg per day, as the patient was not taking statins or any medication related to the side effect of myositis (Patient 6). In this patient, the AST level normalized rapidly, and clinically-acceptable tolerability to daptomycin was observed. Otherwise, no eosinophilic pneumonia was noted in our patients, but we are aware that this is a potentially deadly complication if not well-managed.

To date, the development of resistance to daptomycin of *Staphylococcus aureus* has been a concern. A number of factors are associated with loss of daptomycin susceptibility in *Staphylococcus aureus*. A recent review identified 62 clinical cases in 36 case reports in which daptomycin resistance was observed. In that review, 40 cases occurred after glycopeptide therapy and 15 after vancomycin and/or daptomycin therapy [34]. Another study demonstrated that under a daptomycin dose of <6 mg/kg per day, previous use of teicoplanin and a longer treatment duration were potential risk factors for decreased susceptibility to daptomycin [35]. The mechanism might be due to alterations of the bacterial cell membrane and cell wall [36].

## Conclusion

In our practice, daptomycin combined with suitable surgical intervention had a high success rate in treating resistant staphylococcal PJI. Daptomycin could be a treatment option for patients with these infections, especially in those with chronic kidney disease or intolerance to glycopeptide antibiotics. Further prospective randomized comparative study is needed in the future. Otherwise, we should pay attention to potential serious adverse events and monitor the serum liver enzyme and CPK levels closely.

## Abbreviations

AST: Serum aspartate transaminase
CKD: Chronic kidney disease
CPK: Serum creatine phosphokinase
CRP: C-reactive protein
DAIR: Debridement, antibiotics, irrigation, prosthesis retention
DAP: Daptomycin
eGFR: Estimated glomerular filtration rate
ESR: Erythrocyte sedimentation rate
MDR: Multi-drug-resistant
MIC: Minimum inhibitory concentration
MRCoNS: Methicillin-resistant coagulase-negative Staphylococci
MRSA: Methicillin-resistant *Staphylococcus aureus*
PJI: Periprosthetic joint infection

## References

[1] Cataldo MA, Petrosillo N, Cipriani M, Cauda R, Tacconelli E (2010). Prosthetic joint infection: recent developments in diagnosis and management. J Inf Secur.

[2] Widmer AF (2001). New developments in diagnosis and treatment of infection in orthopedic implants. Clin Infect Dis.

[3] Otto M (2006). Bacterial evasion of antimicrobial peptides by biofilm formation. Curr Top Microbiol Immunol.

[4] Parvizi J, Azzam K, Ghanem E, Austin MS, Rothman RH (2009). Periprosthetic infection due to resistant staphylococci: serious problems on the horizon. Clin Orthop Relat Res.

[5] Tsukayama DT, Goldberg VM, Kyle R (2003). Diagnosis and management of infection after total knee arthroplasty. J Bone Joint Surg Am.

[6] Lichstein P, Su S, Hedlund H, Suh G, Maloney WJ, Goodman SB, Huddleston JI (2016). Treatment of Periprosthetic knee infection with a two-stage protocol using static spacers. Clin Orthop Relat Res.

[7] Costerton JW (2005). Biofilm theory can guide the treatment of device-related orthopaedic infections. Clin Orthop Relat Res.

[8] Sia IG, Berbari EF, Karchmer AW (2005). Prosthetic joint infections. Infect Dis Clin N Am.

[9] Salgado CD, Dash S, Cantey JR, Marculescu CE (2007). Higher risk of failure of methicillin-resistant *Staphylococcus aureus* prosthetic joint infections. Clin Orthop Relat Res.

[10] Lodise TP, Graves J, Evans A, Graffunder E, Helmecke M, Lomaestro BM, Stellrecht K (2008). Relationship between vancomycin MIC and failure among patients with methicillin-resistant *Staphylococcus aureus* bacteremia treated with vancomycin. Antimicrob Agents Chemother.

[11] Appelbaum PC (2007). Reduced glycopeptide susceptibility in methicillin-resistant *Staphylococcus aureus* (MRSA). Int J Antimicrob Agents.

[12] Benvenuto M, Benziger DP, Yankelev S, Vigliani G (2006). Pharmacokinetics and tolerability of daptomycin at doses up to 12 milligrams per kilogram of body weight once daily in healthy volunteers. Antimicrob Agents Chemother.

[13] Tasse J, Croisier D, Badel-Berchoux S, Chavanet P, Bernardi T, Provot C, Laurent F. Preliminary results of a new antibiotic susceptibility test against biofilm installation in device-associated infections: the Antibiofilmogram(R). Pathog Dis. 2016;74(6) 10.1093/femspd/ftw057 27316688

[14] Traunmüller F, Schintler MV, Metzler J, Spendel S, Mauric O, Popovic M, Konz KH, Scharnagl E, Joukhadar C (2010). Soft tissue and bone penetration abilities of daptomycin in diabetic patients with bacterial foot infections. J Antimicrob Chemother.

[15] Kullar R, McClellan I, Geriak M, Sakoulas G (2014). Efficacy and safety of daptomycin in patients with renal impairment: a multicenter retrospective analysis. Pharmacotherapy.

[16] Corona Pérez-Cardona PS, Barro Ojeda V, Rodriguez Pardo D, Pigrau Serrallach C, Guerra Farfán E, Amat Mateu C, Flores SX (2012). Clinical experience with daptomycin for the treatment of patients with knee and hip periprosthetic joint infections. J Antimicrob Chemother.

[17] Byren I, Rege S, Campanaro E, Yankelev S, Anastasiou D, Kuropatkin G, Evans R (2012). Randomized controlled trial of the safety and efficacy of Daptomycin versus standard-of-care therapy for management of patients with osteomyelitis associated with prosthetic devices undergoing two-stage revision arthroplasty. Antimicrob Agents Chemother.

[18] National Kidney Foundation (2002). K/DOQI clinical practice guidelines for chronic kidney disease: evaluation, classification, and stratification. Am J Kidney Dis.

[19] Kuo FC, Yen SH, Peng KT, Wang JW, Lee MS (2016). Methicillin-resistant *Staphylococcal* periprosthetic joint infections can be effectively controlled by systemic and local daptomycin. BMC Infect Dis.

[20] Osmon DR, Berbari EF, Berendt AR, Lew D, Zimmerli W, Steckelberg JM, Rao N, Hanssen A, Wilson WR (2013). Infectious Diseases Society of America. Diagnosis and management of prosthetic joint infection: clinical practice guidelines by the Infectious Diseases Society of America. Clin Infect Dis.

[21] Rao N, Regalla DM (2006). Uncertain efficacy of daptomycin for prosthetic joint infections: a prospective case series. Clin Orthop Relat Res.

[22] Gonzalez-Ruiz A, Beiras-Fernandez A, Lehmkuhl H, Seaton RA, Loeffler J, Chaves RL (2011). Clinical experience with daptomycin in Europe: the first 2.5 years. J Antimicrob Chemother.

[23] Prendki V, Zeller V, Passeron D, Desplaces N, Mamoudy P, Stirnemann J, Marmor S, Ziza JM (2014). Outcome of patients over 80 years of age on prolonged suppressive antibiotic therapy for at least 6 months for prosthetic joint infection. Int J Infect Dis.

[24] Mittal Y, Fehring TK, Hanssen A, Marculescu C, Odum SM, Osmon D (2007). Two-stage reimplantation for periprosthetic knee infection involving resistant organisms. J Bone Joint Surg Am.

[25] Panwar B, Johnson VA, Patel M, Balkovetz DF (2013). Risk of vancomycin-induced nephrotoxicity in the population with chronic kidney disease. Am J Med Sci.

[26] Tongsai S, Koomanachai P (2016). The safety and efficacy of high versus low vancomycin trough levels in the treatment of patients with infections caused by methicillin-resistant *Staphylococcus aureus*: a meta-analysis. BMC Res Notes.

[27] Frank JM, Kayupov E, Moric M, Segreti J, Hansen E, Hartman C (2017). The mark Coventry, MD, award: oral antibiotics reduce reinfection after two-stage exchange: a multicenter, randomized controlled trial. Clin Orthop Relat Res.

[28] Antony S, Tiscareno-Grajeda I, Misenhiemer G, Heyderman J. Use of Daptomycin in the treatment of prosthetic joint infections a prospective observational study of 30 patients with infected prosthetic joint infections. The internet J Infect Dis. 2009;7(1)

[29] Antony SJ, Angelos E, Stratton CW (2006). Clinical experience with Daptomycin in patients with orthopedic-related infections. Infect Dis Clin Pract.

[30] Lora-Tamayo J, Parra-Ruiz J, Rodríguez-Pardo D, Barberán J, Ribera A, Tornero E (2014). High doses of daptomycin (10 mg/kg/d) plus rifampin for the treatment of staphylococcal prosthetic joint infection managed with implant retention: a comparative study. Diagn Microbiol Infect Dis.

[31] Kullar R, Davis SL, Levine DP, Zhao JJ, Crank CW, Segreti J, Sakoulas G, Cosgrove SE, Rybak MJ (2011). High-dose daptomycin for treatment of complicated gram-positive infections: a large, multicenter, retrospective study. Pharmacotherapy.

[32] Ferrera C, Vilacosta I, Vivas D, Olmos C. Severe daptomycin-induced myopathy. Med Clin (Barc). 2012;139(3):138–9. 10.1016/j.medcli.2011.12.001 22285497

[33] Berg ML, Estes LL, Dierkhising RA, Curran B, Enzler MJ (2014). Evaluation of impact of statin use on development of CPK elevation during daptomycin therapy. Ann Pharmacother.

[34] Stefani S, Campanile F, Santagati M, Mezzatesta ML, Cafiso V, Pacini G (2015). Insights and clinical perspectives of daptomycin resistance in *Staphylococcus aureus*: a review of the available evidence. Int J Antimicrob Agents.

[35] Bassetti M, Villa G, Ansaldi F, De Florentiis D, Tascini C, Cojutti P, Righi E, Sartor A, Crapis M, De Rosa FG, Pea F, Menichetti F (2015). Risk factors associated with the onset of daptomycin non-susceptibility in *Staphylococcus aureus* infections in critically ill patients. Intensive Care Med.

[36] Moise PA1, North D, Steenbergen JN, Sakoulas G. Susceptibility relationship between vancomycin and daptomycin in *Staphylococcus aureus*: facts and assumptions. Lancet Infect Dis 2009;9(10):617−624. 10.1016/S1473-3099(09)70200-2 19778764

